# Microbial Terroir in Chilean Valleys: Diversity of Non-conventional Yeast

**DOI:** 10.3389/fmicb.2016.00663

**Published:** 2016-05-17

**Authors:** Carla Jara, V. Felipe Laurie, Albert Mas, Jaime Romero

**Affiliations:** ^1^Facultad de Ciencias Agronómicas, Universidad de Chile, SantiagoChile; ^2^Facultad de Ciencias Agrarias, Universidad de Talca, TalcaChile; ^3^Departament de Bioquímica I Biotecnologia, Facultat d’Enologia, Universitat Rovira i Virgili, TarragonaSpain; ^4^Laboratorio de Biotecnología, Instituto de Nutrición y Tecnología de los Alimentos, Universidad de Chile, SantiagoChile

**Keywords:** wine, non-*Saccharomyces*, *Hanseniaspora*, *Metschnikowia*, *Torulaspora*

## Abstract

In this study, the presence of non-conventional yeast associated with vineyards located between latitudes 30°S and 36°S was examined, including the valleys of Limarí, Casablanca, Maipo, Colchagua, Maule, and Itata. The microbial fingerprinting in each valley was examined based on the specific quantification of yeast of enological interest. Grape–berries were sampled to evaluate the presence and load of non-conventional yeast with enological potential, such as *Metschnikowia*, *Hanseniaspora*, *Torulaspora*, *Debaryomyces*, *Meyerozyma*, and *Rhodotorula*. These yeasts were present in all vineyards studied but with varying loads depending on the valley sampled. No identical fingerprints were observed; however, similarities and differences could be observed among the microbial profiles of each valley. A co-variation in the loads of *Metschnikowia* and *Hanseniaspora* with latitude was observed, showing high loads in the Casablanca and Itata valleys, which was coincident with the higher relative humidity or rainfall of those areas. Non-conventional yeasts were also isolated and identified after sequencing molecular markers. Potentially good aromatic properties were also screened among the isolates, resulting in the selection of mostly *Metschnikowia* and *Hanseniaspora* isolates. Finally, our results suggest that microbial terroir might be affected by climatic conditions such as relative humidity and rainfall, especially impacting the load of non-conventional yeast. In this study, the microbial fingerprint for yeast in Chilean vineyards is reported for the first time revealing an opportunity to study the contribution of this assembly of microorganisms to the final product.

## Introduction

Grape–berries are a great reservoir for microorganisms such as yeasts, lactic acid bacteria, and acetic acid bacteria. Yeasts play a fundamental role in the process of alcoholic fermentation because they are responsible for the transformation of sugars into ethanol, carbon dioxide, and other metabolites (Ribereau-Gayon et al., 2006). Due to their role in alcoholic fermentation, in enology, yeasts are usually divided into two categories: *Saccharomyces* and non-*Saccharomyces*. The latter category includes a wide array of different genera, also termed non-conventional yeasts. *Saccharomyces* has a high fermentative capacity and predominates during alcoholic fermentation (Ribereau-Gayon et al., 2006), whilst non-conventional yeasts proliferate during the first stage of spontaneous fermentation as they can tolerate low concentrations of ethanol, approximately 4% v/v (Fleet, 1993).

Non-conventional yeasts are relevant for their ability to influence the varietal flavors of wines by transforming non-volatile compounds into volatile aromas through enzymatic action (Carrau et al., 2005; Varela et al., 2009; Ciani et al., 2010). Additionally, non-conventional yeasts might influence the fermentative flavor by generating metabolites derived from the fermentation processes (Fleet, 2003; Cordente et al., 2012). Moreira et al. (2008) examined the role of *Hanseniaspora guilliermondii* and *H. uvarum* in pure and mixed starter cultures with *Saccharomyces cerevisiae*. Their results showed that the growth of those yeasts during the early days of fermentation enhanced the production of desirable compounds, such as esters, and had no negative influence through the production of undesirable compounds. Viana et al. (2008) investigated 38 yeast strains, including the *Candida, Hanseniaspora, Pichia, Torulaspora*, and *Zygosaccharomyces* genera, for acetate ester formation. They identified *H. osmophila* as a good candidate for mixed cultures because this yeast has a glycophilic nature, the ability to produce acetaldehyde within a range compatible for wine and acetate ester production. Medina et al. (2013) evaluated the use of a native *H vineae* in Chardonnay must. They found that the aroma sensory analysis indicated a significant increase in fruit intensity, described as banana, pear, apple, citric fruits, and guava, when *H. vineae* was used.

On the grape surface, Fleet (2003) reported very low loads of *Saccharomyces cerevisiae*, at 10^2^ CFU/g of grape–berry, while Beltran et al. (2002) reported that only in very healthy grapes could they recover *S. cerevisiae* in the must. Instead, high loads of non-conventional yeasts ranging from 10^6^–10^8^ CFU/g in grape–berries have been reported several times (Constantí et al., 1997; Torija et al., 2001; Barata et al., 2012). Using Next Generation Sequencing, Setati et al. (2012) and Bokulich et al. (2014) added that non-conventional yeasts on grape–berry, that is, the majority component of the microbiome, will have a potential influence on the organoleptic quality of wine and can even be considered the “microbial terroir” (Gilbert et al., 2014).

In Chile, most vineyards are located between latitudes 30°S and 36°S, along a longitudinal stretch of ca. 1300 km that offers a wide variety of climatic conditions and hence a variety of viticultural areas that may influence the microbial terroir, thus potentially contributing to the distinctive organoleptic properties of wine from each region. To the best of our knowledge, no studies on yeast diversity have been conducted in Chilean vineyards covering a wide range of climate conditions. Therefore, the aim of this work was to understand the diversity and geographic distribution of the microbial communities associated with grape–berries in Chilean valleys. Culture independent approach based on qPCR and DNA extracted directly from grapevines, was used to study the presence and load of yeast of enological interest. This was complemented with the isolation, identification, and characterization of non-conventional yeasts that were also performed to explore the enological potential of native isolates.

## Materials and Methods

### Sampling and Culture Conditions

Grape–berry samples from healthy vines were obtained from several vineyards located in different Chilean valleys between March and May of 2015 (harvest period) with sugar content around 23° Brix. The vineyards were located between latitudes 30°S and 36°S, representing the Limarí, Casablanca, Maipo, Colchagua, Maule, and Itata valleys. Approximately 1 kg of grapes was obtained from at least five plants of each vineyard, as detailed in **Table 1**.

**Table 1:** Vineyard locations and grape cultivars sampled.

Approximate vineyard location	Valleys	Grape cultivar’s
30°39′S–71°19′W	Limarí	Chardonnay
		Pinot Noir
		Muscat of Alexandria
		Carmenere
		Merlot
33°21′S–71°20′W	Casablanca	Pinot noir
		Sauvignon Blanc
		Chardonnay
		Merlot
33°34′S–70°38′W	Maipo	Malbec
		Sauvignon Blanc
		Cabernet Sauvignon
		Carmenere
		Cabernet Franc
		Carmenere
34°39′S–71°12′W	Colchagua	Merlot
		Cabernet Sauvignon
		Cabernet Franc
		Cabernet Sauvignon
35°50′S–71°75′W	Maule	Petit Verdot
		Alicante Bouschet
		Torontel
		Mencia
36°30′S–74°42′W	Itata	País
		Cinsault

The samples were loaded into sterile Stomacher^®^ bags and transported to the laboratory in a coolbox containing ice pads. Once in the laboratory, each sample was transferred into a fresh Stomacher^®^ bag, in which the berries were pressed by hand for 3 min. Then, 5 mL of juice was separated for culture analysis, while 5 mL was centrifuged at 12.000 × *g* for 15 min, and the pellets obtained were frozen at -20°C until DNA extraction.

### Yeast Isolation

Samples for the isolation and identification of yeasts were taken from the juice samples described above. Several decimal dilutions (10^-2^ to 10^-4^) of each sample (0.1 mL) were plated on YEPD agar medium (1% yeast extract, 2% peptone, 2% glucose, and 2% agar by w/v, Merck) with 25 ppm of cycloheximide (Merck) and incubated at 28°C for 2 days. Where possible, 4–6 representatives of each colony-morphology were isolated from plates with ≤200 colonies and purified through two rounds of streak plating onto fresh agar plates. In addition, unique but infrequent colonies that were observed on plates with >200 colonies were also isolated. The isolates were maintained in a cryobank at -80°C.

### DNA Extraction from Grape–Berry Samples and Yeast Isolates

The initial step for our culture independent approach was the extraction of DNA directly from grapevines. The pellets obtained after grape juice centrifugation were re-hydrated with 480 μL Phosphate-buffered saline (PBS), with vigorous agitation. A 20 μL aliquot of 20 mg/mL lyticase (Sigma) was added to the samples, which were subsequently incubated at 37°C for 20 min. Then, the samples were treated with 2.5 μL volume of 20 mg/mL Proteinase K (Merck) incubated at 37°C for 45 min. The Power Soil DNA Isolation Kit (Mo-Bio Laboratories, Inc.) was used for DNA extraction according to the manufacturer’s instructions.

In the case of yeast isolates, each of the colonies selected was suspended in 200 μL PBS, with vigorous agitation, followed by centrifugation at 5.000 × *g* for 5 min. The pellets formed were washed with TE-NaCl (Tris 10mM pH7, EDTA 1 mM, NaCl 0.15 M) and centrifuged at 5.000 × *g* for 5 min. Subsequently, a 20 μL volume of 20 mg/mL lyticase (Sigma) was added to the samples, which were subsequently incubated at 37°C for 20 min. Finally, the samples were treated with 2.5 μL volume of 20 mg/mL Proteinase K (Merck) incubated at 37°C for 45 min. The Power Soil DNA Isolation Kit (Mo-Bio Laboratories, Inc.) was used for DNA extraction according to the manufacturer’s instructions. All the DNA obtained was froze at -20°C until processed.

### Identification of Yeast Isolates

The identification of yeast isolates (non-conventional yeast and *Saccharomyces*) were done by ITS 5.8S rRNA and D1/D2 sequence. The ITS 5.8S-rRNA were amplified using primers ITF1 (5′-TCCGTAGGTGAACCTGCGG-3′) and ITF4 (5′-TCCTCCGCTTATTGATATGC-3′; Esteve-Zarzoso et al., 1999). The partial 26S-rRNA gene sequences (D1/D2 domains) were amplified using primers NL-1 (5′-GCATATCAATAAGCGGAGGAAAAG-3′) and NL-4 (5′-GGTCCGTGTTTCAA GACGG-3′; Kurtzman and Robnett, 1998). DNA sequencing was performed by Macrogen (USA). Also, a BLAST (Basic Alignment Search Tool) analysis^1^ was performed for the sequences obtained. The identification of each isolate was performed based on the closest relative sequence found in the database (GenBank). Isolates were identified and respective sequences were deposited in GenBank (KU350312–KU350496).

### Primer Design

Primers were designed by aligning of the variable D1/D2 domains of the 26S rRNA gene sequences from different yeast species. Sequences were obtained from the GenBank database and alignment was performed with the Clustal W multiple sequence alignment. The final selection of the primers was performed using the Primer-Blast program^2^. A BLAST search was used to check the specificity of each primer as described in **Table 2**.

**Table 2:** Primers and programs for quantitative PCR.

	Programs	Primer	Sequences 5′–3′	Reference
Total yeast	95°C 10 s; 60°C 10 s; 72°C 15 s. Cycle: 50	NL1	GCATATCAATAAGCGGAGGAAAAC	Mills et al., 2002
		NL4	GGTCCGTGTTTCAAGACGG	
*Saccharomyces*	95°C 10 s; 60°C 10 s; 72°C 10 s. Cycle: 35	Sac F	GAAAACTCCACAGTGTGTTG	
		Sac R	GCTTAAGTGCGCGGTCTTG	
*Aureobasidium*	95°C 10 s; 60°C 10 s; 72°C 10 s. Cycle: 40	Aur F	CGCATCGATGAAGAACGCAG	This study
		Aur R	CAACTAAGGACGGCACCCAA	
*Rhodotorula*	95°C 10 s; 60°C 10 s; 72°C 10 s. Cycle: 50	Rho F	ACCTTGCGCTCCTTGGTATT	This study
		Rho R	TCCTTTAACCCAACTCGGCT	
*Meyerozyma*	95°C 10 s; 60°C 10 s; 72°C 10 s. Cycle: 40	Mey F	AGATAGGTTGGGCCAGAGGT	This study
		Mey R	GCATTTCGCTGCGTTCTTCA	
*Torulaspora*	95°C 10 s; 60°C 10 s; 72°C 10 s. Cycle: 40	Tor F	CAAAGTCATCCAAGCCAGC	This study
		Tor R	TTCTCAAACAATCATGTTTGGTAG	
*Metschnikowia*	95°C 10 s; 60°C 10 s; 72°C 10 s. Cycle: 40	Met F	CAACGCCCTCATCCCAGA	This study
		MetR	AGTGTCTGCTTGCAAGCC	
*Debaryomyces*	95°C 10 s; 57°C 10 s; 72°C 10 s. Cycle: 50	Deb F	TGAAGAACGCAGCGAAATGC	This study
		Deb R	GCCGAGCCTAGAATACCGAG	
*Hanseniaspora*	95°C 10 s; 57°C 10 s; 72°C 10 s. Cycle: 50	HanF	CCCTTTGCCTAAGGTACG	This study
		HanR	CGCTGTTCTCGCTGTGATG	

### Quantitative PCR (qPCR) and Standard Curves

Specific qPCR reactions were carried out to examine the presence and load of yeast of enological interest. The qPCR reactions were performed using an AriaMx real-time PCR System (Agilent Technologies) using primers and programs described in **Table 2**, for the following yeast: *Saccharomyces, Hanseniaspora, Torulaspora, Metschnikowia, Rhodotorula, Debaryomyces, and Meyerozyma.* Standard curves were built for each yeast species in triplicate using 10-fold serial dilutions of fresh cultures.

PCR amplification was performed in 10 μL of mix containing 1 μL of DNA 0.5 pmol/μL of each respective primer, 8 μL of LightCycler 480 SYBR Green I Master (Roche) and 1 μL of Milli-Q sterile H_2_O. All of the amplifications were carried out in optical-grade, 96-well plates, AriaMx real-time PCR System (Agilent Technologies). All samples were analyzed in triplicate. Yeast load were compared using grouped analysis performed with GraphPad Prism version 6.00 for Mac (GraphPad Software, La Jolla CA, USA^3^).

### Phylogenetic Analysis

The nucleotide sequences of the 5.8S ITS rDNA region and D1/D2 domain part gen 26S rRNA were compared with those available in the GenBank database using the BLAST method in order to investigate their approximate phylogenetic affiliation, and their sequence similarities were determined at the National Center for Biotechnology Information, USA (Altschul et al., 1997)^4^. Phylogenetic and molecular evolutionary analyses were performed using MEGA software, version 6.0 Beta. The phylogenetic tree for 5.8S ITS and D1/D2 domain part 26S rRNA gen were constructed by UPGMA (unweighted pair-group method with arithmetic mean) method. The evolutionary distances were computed using the Maximum Composite Likelihood method, using the Mega 6 (version 6.0) software package obtained from the website^5^.

### Laboratory-Scale Fermentations and Yeast Selection

With the yeast isolates obtained from each of the grape–berry samples, a series of micro-fermentations were conducted for an initial assessment of the fermentation capacity and aromatic attributes of the isolates. Fifty milliliters of synthetic must (**Table 3**) supplemented with 20 mg/L of SO_2_ was inoculated to a final concentration of 10^8^ cells/mL. Micro-fermentations were conducted at 18°C with stirring in an orbital shaker at a rate of 150 rpm. The evolution of the alcoholic fermentation was evaluated by monitoring weight loss every two days. At the end of the fermentation, the concentrations of glucose/fructose were measured using enzymatic kits (Boehringer Mannheim), according to the manufacturer’s instructions.

**Table 3:** Synthetic must composition.

Components	
Glucose	100 g
Fructose	100 g
Citric acid	0.5 g
Malic acid	5 g
Tartaric acid	3 g
KH_2_PO_4_	0.750 g
K_2_SO_4_	0.500 g
MgSO_2_ 7 H_2_O	0.250 g
CaCl_2_ 2 H_2_O	0.155 g
NaCl	0.200 g
Nitrogen	408 mg/L
NH_4_Cl (120 mgN/l)	0.460 g
Amino acid stock ***	13.09 mL
Oligo-elements stock *	1 mL
Vitamins stock **	10 mL
Distilled H_2_O	1 L
Vitamins Stocks (for 1 liter)**	
Myo-inositol	2 g
Pantothenate calcium	0.15 g
Thiamine, hydrochloride	0.025 g
Nicotinic acid	0.2 g
Pyridoxine	0.025 g
Biotine	3 mL
Distilled H_2_O	csp 1 L
Oligo-elements stock (1 liter)*	
MnSO_4_, H_2_O	4 g
ZnSO_4_, 7 H_2_O	4 g
CuSO_4_, 5 H_2_O	1 g
KI	1 g
CoCl_2_, 6 H_2_O	0.4 g
H_3_BO_3_	1 g
(NH_4_)_6_MO_7_O_24_	1 g
Distilled H_2_O	
**Stock anaerobiosis factors (100 mL)**	
Ergosterol	1.5 g
Oleic Acid	0.5 mL
Tween 80	50 mL
Ethanol	cps 100 mL
Amino acids stocks (for 1 liter solution Na_2_CO_3_ 2%) ***	
Tyrosine (Tyr)	1.5 g
Tryptophan (Trp)	13.4 g
Isoleucine (Ile)	2.5 g
Aspartic Acid (Asp)	3.4 g
Glutamic Acid (Glu)	9.2 g
Arginine (Arg)	28.3 g
Leucine (Leu)	3.7 g
Threonine (Thr)	5.8 g
Glycine (Gly)	1.4 g
Glutamine (Gln)	38.4 g
Alanine (Ala)	11.2 g
Valine (Val)	3.4 g
Methionine (Met)	2.4 g
Phenylalanine (Phe)	2.9 g
Serine (Ser)	6.0 g
Histidine (His)	2.6 g
Lysine (Lys)	1.3 g
Cysteine (Cys)	1.5 g
Proline (Pro)	46.1 g
Distilled H_2_O	cps 1 L
**Total**	138 g

### Sensory Analyses

The resulting micro-fermentations were evaluated at controlled room temperature (20°C), in individual booths. The sensory analyses were carried out by olfactory evaluation in the Sensory evaluation laboratory and all panelists were winemakers belonging to the Enology Department, Universidad de Chile. Terpenes, thiols, and higher alcohols were represented as aromatic descriptors such as “fruit” and/or “flower” and were order as aromatic groups: fermentative, tropical fruit, citrus fruit, stone fruits, berries, flower, dried fruit, cooked fruits, and sweet aromas. The sensory panel first smelled several fresh aromatic references to choose those samples which best fitted their aroma. The strategy for data analysis was a descriptive method (attribute score versus frequencies of citation). The number of times each attribute was cited as negative frequency and positive frequency for each sample were counted up. Once all data were collected, the list of yeast was ranked according to their citation frequency to identify the most relevant attribute of each fermented product.

### Climate Data

Monthly weather data were extracted from the Agromet INIA^6^ and Red Agroclimática Nacional^7^ databases. Data were collected from seven different weather stations representing each vineyard. Monthly measurements were extracted for average temperature, maximum temperature, minimum temperature, rainfall, and average relative humidity during 2015 (**Figure 2**).

## Results

### Non-conventional Yeast in Different Chilean Valleys and Climatic Conditions

**FIGURE 1:**
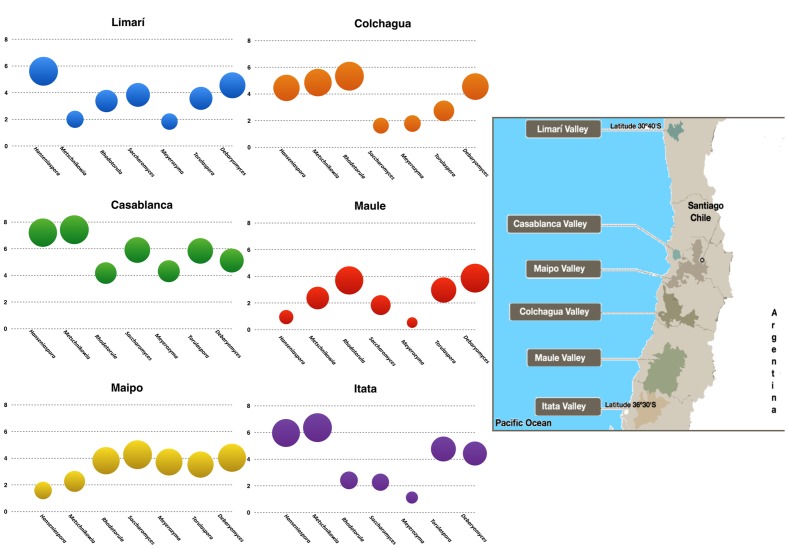
**Microbial terroir in different Chilean Valleys.** The graphics represent the load of the yeast of enological interest as were quantified by qPCR in the different valleys examined (log_10_ scale). The insert in the right corresponds to a map showing the location of the valleys.

**FIGURE 2:**
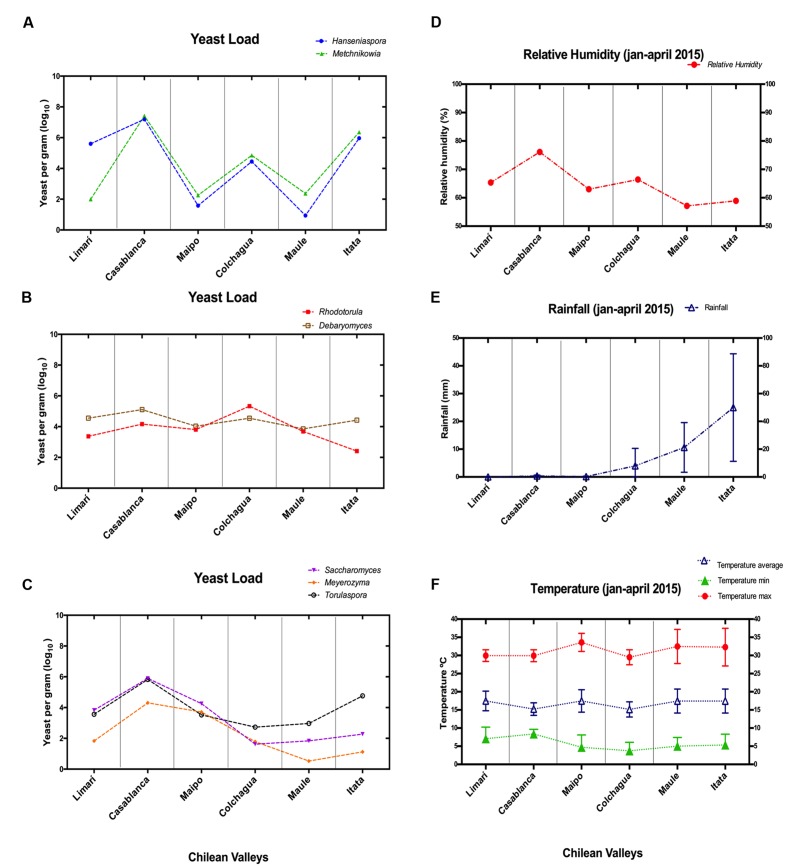
**Non-conventional yeast loads and climatic factors in Chilean valleys. (A–C)** Correspond to qPCR data expressed as yeast per grams (scale log_10_). **(A)** *Hanseniaspora/Metschnikowia*; **(B)** *Torulaspora/Saccharomyces/Meyerozyma*; **(C)** *Rhodotorula/Debaryomyces*. **(D–F)** Correspond to climatic factors. **(D)** Relative humidity (%); **(E)** Rainfall (mm); **(F)** Temperature (Celsius degree).

A total of twenty-five samples of grape–berries from six Chilean viticultural areas were analyzed (**Table 1**), screening for *Saccharomyces* and six non-conventional yeast genera *Torulaspora, Hanseniaspora*, *Metschnikowia, Rhodotorula, Debaryomyces*, and *Meyerozyma*. All of these yeast were present at different loads depending on the examined samples. The yeast population (log_10_ scale) was represented for each valley and it revealed the microbial fingerprint for each area, ordered from north to south (**Figure 1**). No identical fingerprints were observed, however, similarities and differences could be observed among the microbial profiles of each valley. For example, *Hanseniaspora* and *Metschnikowia* were present in Limarí, Casablanca, and Itata valleys at comparatively high loads with respect to Maipo and Maule valleys. Interestingly, the following three sets of yeast showed load patterns that were similar across valleys: *Hanseniaspora* and *Metschnikowia; Torulaspora, Saccharomyces, and Meyerozyma*; *and Rhodotorula and Debaryomyces* (**Figure 2**). The vineyard samples with the highest yeast load were the ones collected in Casablanca Valley, with a dominant presence of *Hanseniaspora* and *Metschnikowia*, with 10^7^ yeasts per gram of fruit. *Torulaspora/Saccharomyces/Meyerozyma* showed a similar population trend along latitude, with high loads in Casablanca (10^5^ yeasts per gram), lower toward the South (about <10^3^ yeasts per gram), with a slight increase for the Itata vineyards. In contrast, the population load of *Rhodotorula* and *Debaryomyces* presented a similar behavior along latitude, maintaining loads between 10^3^ and 10^5^ yeasts per gram of fruit.

To try to explain the differences observed in population patterns depending on vineyard location, the relative humidity, rainfall, and temperatures of these areas were explored (**Figure 2**). The high population loads of *Hanseniaspora*/*Metschnikowia* observed in the Casablanca and Itata valleys were coincident with the highest relative humidity and rainfall observed for those valleys, respectively. Similarly, the load pattern of *Torulaspora/Saccharomyces/Meyerozyma* could also be linked with the types of climate variations. They showed the highest load in valleys with low rainfall (Limarí, Casablanca, and Maipo), but their load was reduced in valleys with high rainfall (Colchagua, Maule, and Itata valleys). Finally, *Rhodotorula/Debaryomyces* seemed to be independent of relative humidity and rainfall, and their load might be linked to the temperatures observed in the valleys.

### Identification of Yeast Isolates

**FIGURE 3:**
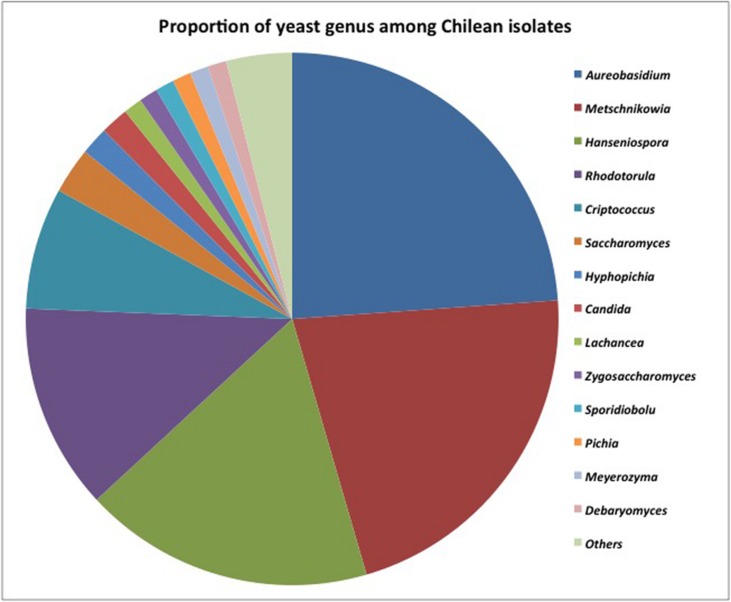
**Distribution of isolates retrieved from different Chilean valleys among yeast genera**.

More than 200 yeasts were isolated, and the results are summarized in **Figure 3**. Among them, 164 different sequences were identified as non-conventional yeasts matching 15 different yeast genera (97.3%) and *Saccharomyces* (2.7%). These results showed an important presence of non-conventional yeast isolates in Chilean valleys. The dimorphic *Aureobasidium* (24%) black yeast-like fungus was widely distributed in the Maipo and Maule valleys. The predominant non-conventional genera were *Metschnikowia* (21%), *Hanseniaspora* (18%), and *Rhodotorula* (13%). Minority genera included *Cryptococcus* (6%), *Hyphopichia* (2%), and *Candida* (2%). Other isolates corresponded to *Lachancea, Zygosaccharomyces, Sporidiobolus, Pichia, Meyerozyma, Debaryomyces, and Torulaspora*, which represented less than 1% as a group.

Among the total samples isolated, 61 yeast sequences were included in a phylogenetic analysis. **Supplementary Figure S1** shows the tree generated by the D1/D2 domain of the partial 26S rRNA sequences from *Metschnikowia* isolates, in which 16 different phylotypes can be observed, indicating the high genetic diversity of *Metschnikowia*. On the other hand, the analysis of *Rhodotorula* and *Hanseniaspora* based on D1/D2 sequences (**Supplementary Figure S2**) showed limited genetic diversity, with four phylotypes for *Rhodotorula* and six for *Hanseniaspora*. The observed diversity of phylotypes seems to be randomly distributed among vineyards rather than corresponding to the predominance of specific genotypes depending on the geographical areas.

### Selection of Isolates Using Micro-fermentations

To select non-conventional yeast with enological potential, all of the strains were tested for their resistance to SO_2_. Micro-fermentations were performed in synthetic must, which was supplemented with metabisulfite to address this criterion. Only 106 isolates were able to tolerate metabisulfite. Then, the enological potential of these isolates was evaluated based on their ability to produce desirable aromas during micro-fermentation.

The attributes most frequently observed in the micro-fermentations were those belonging to the fruity, flowery, and fermentative aromas. Nineteen non-conventional yeasts were selected based on descriptive aromatic profiles (**Table 4**). These isolates corresponded to *Metschnikowia* (Casablanca and Itata valleys*), Hanseniaspora* (Maipo Valley*), Rhodotorula* (Limarí and Maipo valleys), *Hyphopichia* (Maipo and Itata valleys), *Candida* (Maule valley), *Lachancea* (Limarí valley), *Pichia* (Maipo valley), *Debaryomyces* (Maipo Valley), and *Citeromyces* (Limarí valley). The main aromatic attribute in *Debaryomyces* was dried fruits; in *Hanseniaspora* isolates were stone, tropical fruits, and sweet aromas; in *Pichia* isolates were tropical fruits and berries. Interestingly, several *Metschnikowia* isolates offered different attributes such as fermentative and sweet aromas, tropical, and stone fruits.

**Table 4:** Sensory description evalutaion and residual sugar from select isolates.

Isolates	Identification	Aromatic groups	Frequency of citation	Residual sugar g/L
PS PN2	*Candida*	Flowers/sweet aromas	100%	143.4
CHLI 14	*Citeromyces*	Fermentative aromas	66,6%	76.1
CARHP5	*Debaryomyces*	Dried fruits	100%	133.3
SB1 MP3	*Hanseniaspora*	Stone fruits/Tropical fruits	100%	74.0
CarLi 3C 1	*Hanseniaspora*	Tropical fruits	100%	21.5
SB2 MP1	*Hanseniaspora*	Stone fruits	100%	9.7
MALI66N 1	*Hanseniaspora*	Sweet aromas	83,3%	21.1
PS MG7	*Hyphopichia*	Cooked fruits	83,3%	84.2
CS AN2	*Hyphopichia*	Sweet aromas	100%	130.3
PNLI 29	*Lachancea*	Fermentative aromas	66,6%	77.8
ML CB1	*Metschinikowia*	Sweet aromas	83,3%	75.4
PN CB1	*Metschinikowia*	Sweet aromas	100%	85.8
PS MG4	*Metschinikowia*	Tropical fruits	100%	102.4
PS MG2	*Metschinikowia*	Stone fruits	100%	75.1
SB3 MP5	*Metschinikowia*	Sweet aromas	83,3%	142.2
SB2 MP5	*Pichia*	Tropical fruits/Berry fruits	100%	125.6
SB2 MP6	*Pichia*	Tropical fruits	100%	136.1
MB AN8	*Rhodotorula*	Flowers	100%	146.7

The fermentation abilities of the isolates were also tested. **Table 4** shows the residual sugar concentration at the end of the micro-fermentation. Some isolates such *Hanseniaspora* consumed almost all the sugar, while others consumed an intermediate range (*Metschnikowia*, *Lachancea*, and *Citeromyces*). Other isolates can be considered as poor fermenters, with less than 50% sugar consumption.

## Discussion

This study evaluated the load and diversity of non-conventional yeasts in Chilean vineyards and was the starting point of a study whose final goal is take advantage of the native microbiome by selection of local strains with interesting enological properties. Similarly to the animal microbiome, the plant microbiome may have important roles for their host, such as improving the availability of organic matter and preventing the growth pathogens through competition for space and nutrients (Gilbert et al., 2014). Studying the microbial ecology in the context of viticulture and wine, offers the opportunity to discover the denominated “microbial terroir” and the contribution of this assembly of microorganisms to the whole process and the final product. The microbial fingerprint for yeast in the Chilean vineyards has not been previously reported.

This study has covered a wide viticultural region (from 30°S to 36°S latitude approximately) that included the most important Chilean valleys and described the microbial fingerprinting based on the yeast of enological interest by using a culture independent approach. In contrast, Bokulich et al. (2014) used a high-throughput, short-amplicon sequencing approach to test the regional distribution of fungal and bacterial communities associated to vineyards in Napa and Sonoma valleys, which are located in the same latitude, covering from N 38°6.8′ to N 38°50.40′ approximately. The next generation sequencing approach allowed a deep examination of microbial communities (Bokulich et al., 2014) and detection of the differences between these close locations (Gilbert et al., 2014). Using a simpler approach to cover more distant locations (Hierro et al., 2006), we were able to establish that grape-surface microbial communities were different among Chilean regions. These differences in the microbial profiles may be related to climatic factors, as northern and southern regions of Chile present important discrepancies in the wheatear conditions.

Additionally, our results allowed us to identify co-variations in the loads of *Metschnikowia*/*Hanseniaspora, Torulaspora/Saccharomyces/Meyerozyma*, and *Rhodotorula/Debaryomyces* that were observed along latitude and associated with relative humidity, rainfall and temperature, respectively. These observations are consistent with studies by Gayevskiy and Goddard (2012), reporting that the proportions of these non-conventional yeasts varied in each sampling zone. Several authors attribute these changes to the geographical location and climatic conditions of the vineyard (Parish and Carroll, 1985; Longo et al., 1991), and it has also been suggested that rainfall could be among the most important factors affecting the load of non-conventional yeasts (Rousseau and Doneche, 2001). Combina et al. (2005), reported that rain near harvest can induce changes in yeast populations, affecting *Metschnikowia* and *Hanseniaspora*. Similarly, Itata valley showed the highest rainfall during the harvest period, which coincided with a high load of *Metschnikowia*/*Hanseniaspora*. Recent studies by Brilli et al. (2014) showed the influence of relative humidity on non-conventional yeast populations, indicating that an increase in relative humidity might induce higher loads of non-conventional yeast during grape ripening. Similar studies by Van der Westhuizen et al. (2000a,b), confirmed that yeast loads observed in coastal areas were higher than in the inland area. Therefore, those studies support the idea that higher load of *Metschnikowia*/*Hanseniaspora* in Casablanca valley, located near to the coast, may be related to relative humidity, as this parameter is highest due to the pronounced maritime influence. Those reports showed that rainfall and relative humidity favored a prevalence of *Hanseniaspora* and *Metschnikowia* yeast on the grape–berries, which could explain the co-variation observed in the Chilean valleys. Contrasting results were obtained for *Torulaspora/Saccharomyces/Meyerozyma*, which seems to vary inversely with rainfall. However, these results have not been reported previously and demand more research efforts to define the negative influence of the rainfall on the load of those yeasts.

Based on the culture dependent approach, our results showed that *Hanseniaspora*, *Metschnikowia*, and *Aureobasidium* were the main genera present on grape–berries in all of the vineyards studied in Chile. These observations are consistent with the findings reviewed by Bisson and Joseph (2009) and Barata et al. (2012) establishing that those genera, along with *Candida*, were the main ones present on grape–berries examined in Spain, Canada, and Argentina. Molecular biology techniques provide a simple and rapid method to differentiate yeasts based on their genetic background (Granchi et al., 1998; Torija et al., 2001). The modern taxonomy of yeasts has been improved by molecular biology techniques providing reliable methods to differentiate yeasts based on their genetic background, mainly the phylogenetic analysis of conserved DNA and protein sequences. Repeats of the chromosomal rDNA sequences have been widely used for the identification and barcoding of yeast genera and species. The D1/D2 rDNA sequences are frequently used in the phylogenetic analysis of yeast, where yeast isolates differing by more than 1% substitutions in the D1/D2 domain represent separate species (Kurtzman and Robnett, 1998). The comparison of D1/D2 domains in our *Metschnikowia* isolates from different Chilean valleys showed that the high genetic diversity was related to 26 polymorphic sites that generate 16 phylotypes. These results agreed with the findings of Sipiczki et al. (2013), which reported 18 and 25 substitutions in the D1/D2 domain in *Metschnikowia* species.

Non-conventional yeasts influenced the wine aroma: terpenes, thiols, esters, and higher alcohols are the most typical aromatic compounds that contribute to the enhancement of sweet-fruity aromas in wines (Mason and Dufour, 2000; Clemente-Jimenez et al., 2004; Sumby et al., 2010; Viana et al., 2011; Gobbi et al., 2013; Jolly et al., 2014). Most of the evidence of the effect of non-conventional yeasts has been obtained from co-inoculation studies combining non-conventional yeast and *Saccharomyces* in different wines. Medina et al. (2012) indicated that the inoculation of *Hanseniaspora* isolates in Chardonnay produced a uniquely fruity character, such as banana, pear apple, citric fruits, grape fruit, and guava. *Metschnikowia* has been used in base wine for sparkling wine production, improving the aromatic profile by increasing smoky and flowery notes (González-Royo et al., 2015). In another example, it was reported that *Debaryomyces* increased the concentrations of citronellol, nerol, and geraniol, which resulted in floral and citrus-type aromas in wine (Garcia et al., 2002); however, the *Debaryomyces* isolate obtained in this study showed a different property, improving dried fruit aromas. These studies have demonstrated that non-conventional yeast can be selected based on their ability to produce aromatic secondary metabolites that contribute to improving the quality of wine, which is presently a very important area of applied interest in oenology.

In our study, we focused on the selection of different non-conventional yeasts as a basis for the selection of interesting strains to be used by the wine industry. In our hands, the most promising yeast strains corresponded to the *Hanseniaspora, Metschnikowia*, and *Debaryomyces* genera. Therefore, our isolates might influence the wine quality through the release of several aromatic compounds, such as esters, higher alcohols, acids, and monoterpenes. Thus, further research to analyze the potential of these strains is underway to select new starter presentations for the wine industry. Furthermore, these non-conventional yeasts reveal the potential of the yeast microbiome to contribute to the complexity and typicality of the wine and conferring the aromatic profiles of specific regions. This possibility is another aspect that is currently being developed in our laboratories.

## Author Contributions

Conceived and designed the experiments: CJ, VL, AM, and JR. Performed the experiments: CJ and JR. Generated and analyzed the data: CJ, VL, AM, and JR. Wrote the paper: CJ, VL, AM, and JR.

## Conflict of Interest Statement

The authors declare that the research was conducted in the absence of any commercial or financial relationships that could be construed as a potential conflict of interest.

## Footnotes

1 http://www.ncbi.nlm.nih.gov/blast/Blast.cgi

2 http://www.ncbi.nlm.nih.gov/tools/primer-blast/

3 www.graphpad.com

4 http://ncbi.nlm.nih.gov/BLAST

5 www.megasoftware.net

6 http://agromet.inia.cl

7 www.agromet.cl

## Supplementary Material

FIGURE S1 **Phylogenetic analysis of Chilean of *Metschnikowia* using D1/D2 domain**. Two sequences from GenBank were included as reference (KM249369 and KM658981).

FIGURE S2 **Phylogenetic analysis of Chilean isolates of using D1/D2 domain for *Hanseniaspora* and *Rhodotorula***.
