# Supplementary figures and images for: Microbial Terroir in Chilean Valleys: Diversity of Non-conventional Yeast
Source: Front Microbiol. 2016 May 17;7:663. doi: 10.3389/fmicb.2016.00663 (PMC4868835; doi:10.3389/fmicb.2016.00663)

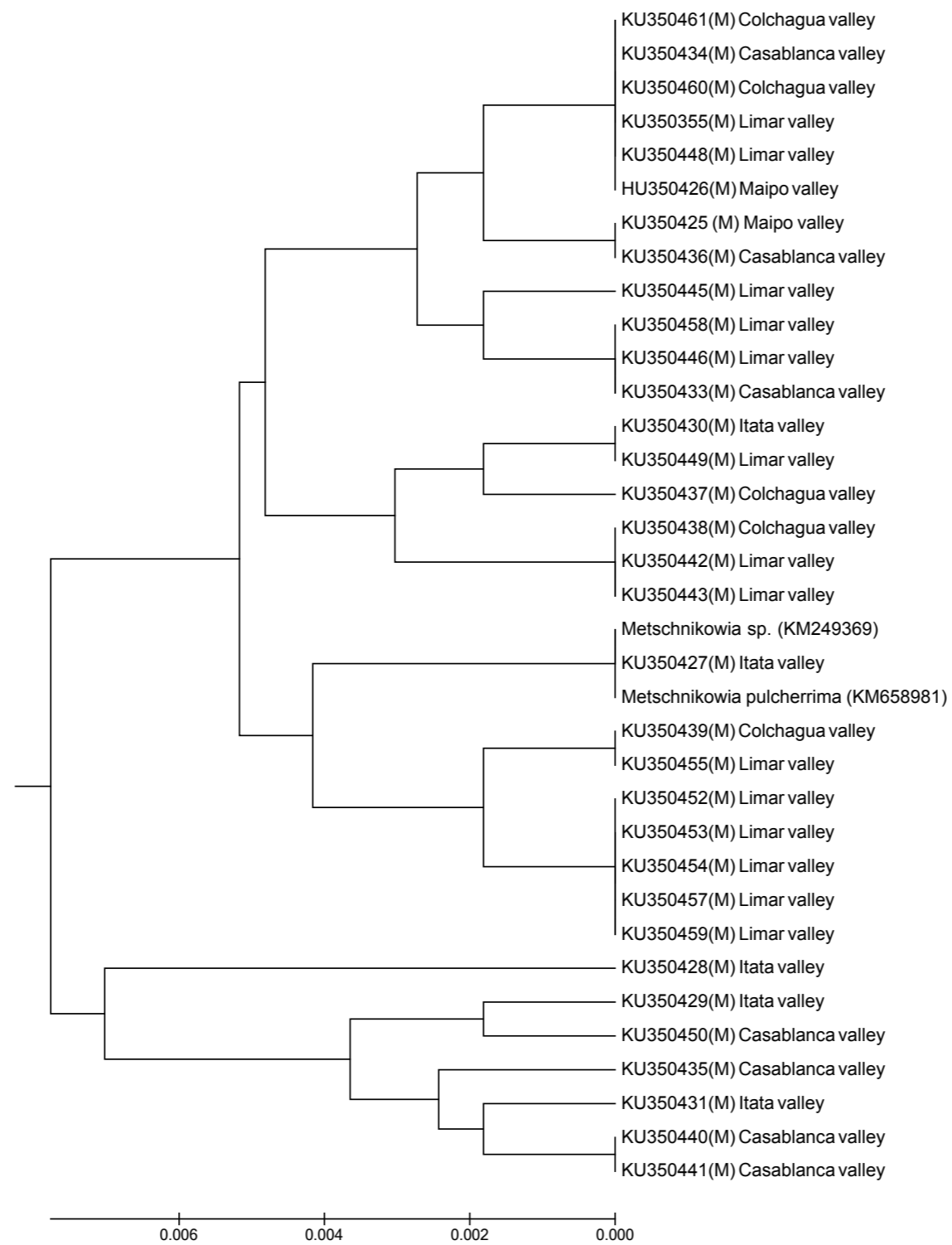

Figure S1

Supplement: FIGURE S1 — Phylogenetic analysis of Chilean of Metschnikowia using D1/D2 domain. Two sequences from GenBank were included as reference (KM249369 and KM658981). [file Presentation_1.PDF]

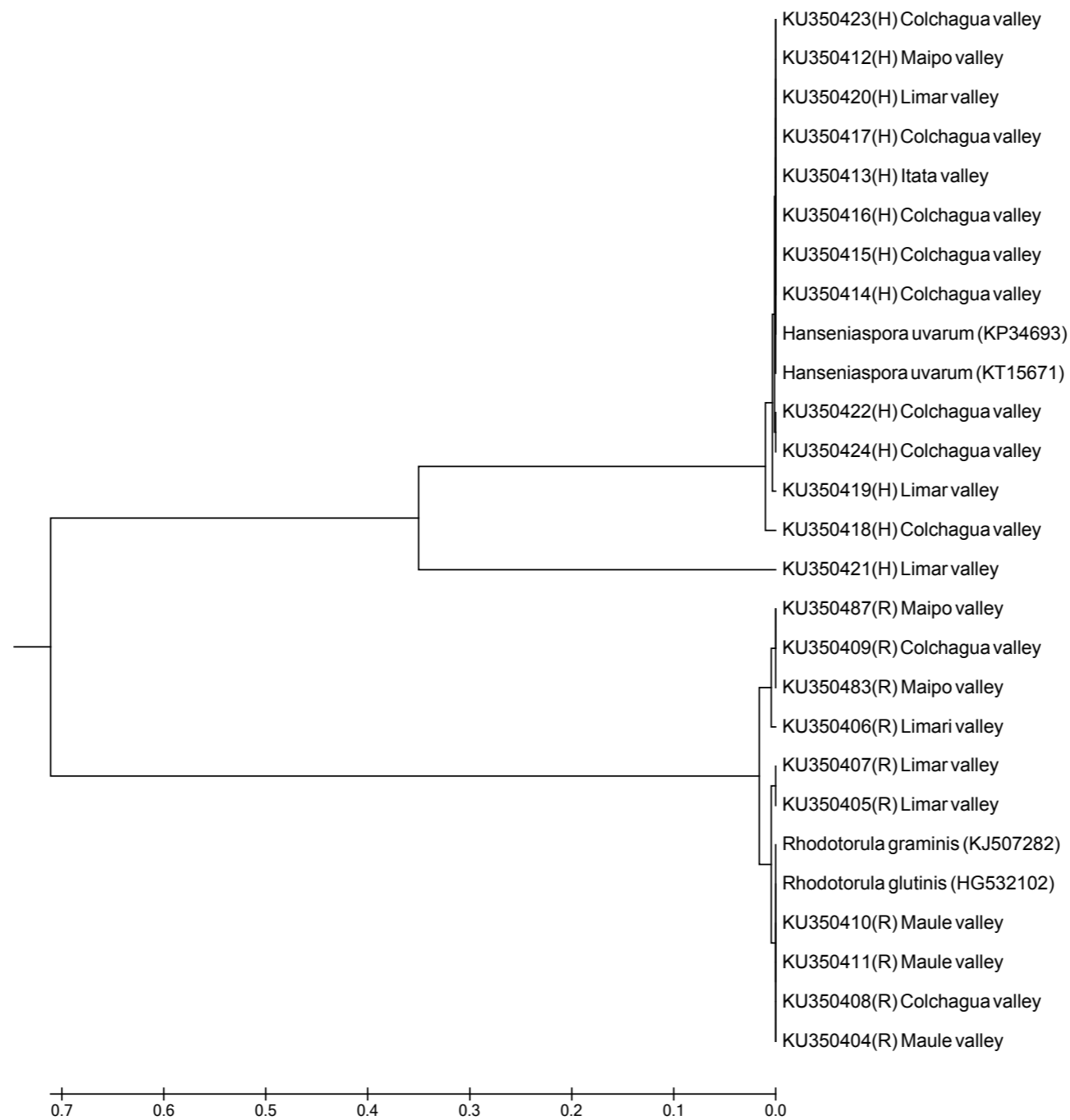

Figure S2

Supplement: FIGURE S2 — Phylogenetic analysis of Chilean isolates of using D1/D2 domain for Hanseniaspora and Rhodotorula. [file Presentation_2.PDF]
